# *Lactobacillus plantarum* CQPC11 Isolated from Sichuan Pickled Cabbages Antagonizes d-galactose-Induced Oxidation and Aging in Mice

**DOI:** 10.3390/molecules23113026

**Published:** 2018-11-20

**Authors:** Yu Qian, Jing Zhang, Xianrong Zhou, Ruokun Yi, Jianfei Mu, Xingyao Long, Yanni Pan, Xin Zhao, Weiwei Liu

**Affiliations:** 1 Chongqing Collaborative Innovation Center for Functional Food, Chongqing University of Education, Chongqing 400067, China; qianyubaby@126.com (Y.Q.); zhouxr@foods.ac.cn (X.Z.); yirk@cque.edu.cn (R.Y.); mujianfei@foods.ac.cn (J.M.); longyaoyao@foods.ac.cn (X.L.); panyanni@foods.ac.cn (Y.P.); 2 Chongqing Engineering Research Center of Functional Food, Chongqing University of Education, Chongqing 400067, China; 3 Chongqing Engineering Laboratory for Research and Development of Functional Food, Chongqing University of Education, Chongqing 400067, China; 4 College of Biological and Chemical Engineering, Chongqing University of Education, Chongqing 400067, China; 5 Environment and Quality Inspection College, Chongqing Chemical Industry Vocational College, Chongqing 401228, China; zjinger0810@126.com; 6 College of Food Science, Southwest University, Chongqing 400715, China; 7 Department of Food Science and Biotechnology, Cha University, Seongnam 13488, Gyeongghi-do, Korea; 8 School of Public Health and Management, Chongqing Medical University, Chongqing 400016, China

**Keywords:** *Lactobacillus plantarum* CQPC11, pickled cabbage, oxidation, aging, mice

## Abstract

Chinese pickled cabbage is a traditional fermented food that contains abundant microbes produced during the process of fermentation. In this work, an in vivo animal study was conducted to investigate the effects of a newly isolated lactic acid bacterium (*Lactobacillus plantarum* CQPC11, LP-CQPC11) on d-galactose-induced oxidation and aging in mice. Analysis of the serum and tissue samples of these mice using molecular biology approaches showed that LP-CQPC11 suppressed the decrease in thymus, brain, heart, liver, spleen, and kidney indices caused by oxidation and aging. Furthermore, LP-CQPC11 increased the levels of SOD (superoxide dismutase), GSH-Px (glutathione peroxidase), and GSH (glutathione), whereas it reduced the levels of NO (nitric oxide) and MDA (malondialdehyde) in the serum, liver, and spleen of oxidation and aging mouse models. Pathological observation indicated that LP-CQPC11 alleviated the damage caused by oxidation and aging on the liver and spleen of mice. qPCR analysis indicated that LP-CQPC11 effectively upregulated the expression of *nNOS* (neuronal nitric oxide synthase), *eNOS* (endothelial nitric oxide synthase), *Cu/Zn-SOD* (cuprozinc-superoxide dismutase), *Mn-SOD* (manganese superoxide dismutase), *CAT* (catalase), *HO-1* (heme oxygenase-1), *Nrf2* (nuclear factor-erythroid 2 related factor 2), γ*-GCS* (γ-glutamylcysteine synthetase), and *NQO1* (NAD(P)H dehydrogenase [quinone] 1), but downregulated the expression of *iNOS* (inducible nitric oxide synthase) in the mouse liver and spleen. Western blot analysis showed that LP-CQPC11 effectively upregulated SOD1 (*Cu/Zn-SOD*), SOD2 (*Mn-SOD*), CAT, GSH1 (c-glutamylcysteine synthetase), and GSH2 (glutathione synthetase) protein expression in mouse liver and spleen tissues. These findings suggest that LP-CQPC11 can effectively prevent d-galactose-induced oxidation and aging in mice, and the effect is even better than that of the commonly used *Lactobacillus delbruechii* subsp. *bulgaricus* (LDSB) and vitamin C in the industry. Thus, LP-CQPC11 may be potentially employed as a probiotic strain.

## 1. Introduction

Chinese Sichuan pickled cabbages are made from fresh vegetables with salt, seasoning, and water through anaerobic fermentation by the naturally occurring flora on the surface of the vegetables. Fermentation occurs in unglazed clay jars that are sealed by cold boiled water [1]. Because the initial brine is often used repeatedly for several years or even decades, the pickling actually represents a long-term screening process that can select a stable flora that is highly diverse and exhibits superior fermentation performance [2]. Many bacterial strains have been isolated from Sichuan pickled cabbages, including *Lactobacillus brevis*, *L. plantarum*, *Pediococcus ethanolidurans*, *L. casei*, *L. pentosus*, *L. sake*, *L. alimentarius*, and *Leuconostoc mesenteroides* [3,4,5]. Particularly during the middle and late stages of fermentation, acid-resistant and non-gas-producing lactic acid bacteria *L. plantarum* and *L. brevis* gradually become the predominant flora in the pickled cabbages [6]. These lactic acid bacteria utilize the soluble nutrients such as sugars and nitrogenous substances in the brine to produce acidic substances as well as other flavor substances, thus giving Sichuan pickled cabbages a unique flavor [7]. These lactic acid bacteria are not only crucial to the quality and flavor of Sichuan pickled cabbages, but are also probiotics that can help maintain human health.

Studies have shown that lactic acid bacteria perform many important physiological functions of the human body and play an important role in maintaining the balance of human microecology. These improve food digestibility and utilization rate in the gastrointestinal tract, decrease serum cholesterol levels, control endotoxin production, inhibit the growth of spoilage bacteria and the generation of food spoilage in the intestine, produce nutrients, and stimulate tissue development, thereby influencing the nutritional status, physiology, infection, pharmacology, toxicology, immune response, tumorigenesis, aging process, and stress responses of the human body [8,9,10,11]. A reduction in the amount of lactic acid bacteria in the human body may impair health. Based on the beneficial effects of lactic acid bacteria, these have been widely used in the food and pharmaceutical industry.

Aging is an inevitable biological process of growing older, which is accompanied by signs of decline in function, adaptability, and disease resistance of the body. Aging is an important risk factor for many diseases, including hypertension, type 2 diabetes, atherosclerosis, and senile dementia, whereas the onset of these diseases can be delayed by slowing down aging [12,13,14]. d-galactose is currently recognized as an aging-inducing agent that can induce aging in animal models, which is similar to the natural aging process, and thus has been widely used in the creation of animal models of aging. A small amount of d-galactose in the body can be converted into glucose and enter the metabolic pathway. However, a large amount of d-galactose may cause cell metabolism disorders and alter the activity of cell oxidases, which in turn produce a large amount of superoxide anions and other oxidation products that damage the structure and function of biological macromolecules, ultimately leading to aging [15]. d-galactose-induced animal models of aging are often used to verify the effects of antioxidants on oxidation and aging before their application to heath care products.

In this study, a strain of lactic acid bacterium was isolated from Sichuan pickled cabbages and was named *L. plantarum* CQPC11 (LP-CQPC11). As a first step toward human studies and to develop health care products, the anti-oxidation-induced aging effects of this strain were assessed in d-galactose-induced mouse models of aging.

## 2. Results

### 2.1. Isolation and Identification of LP-CQPC11

**Figure 1:**
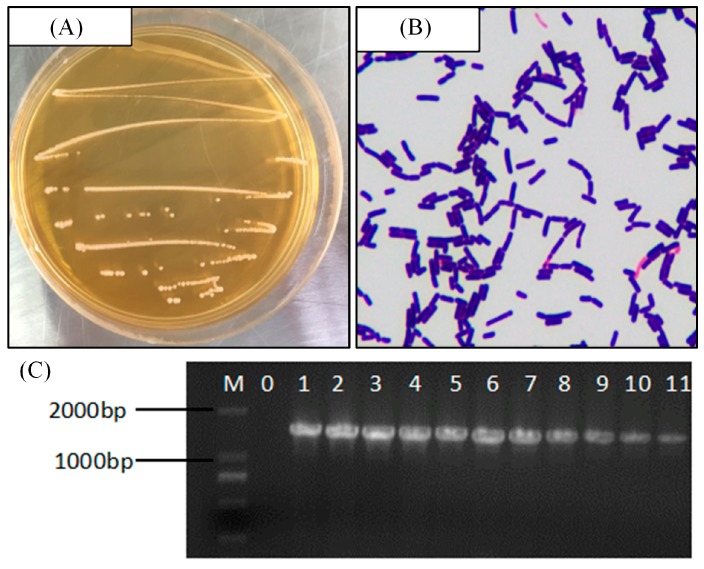
(**A**) Colony morphology, (**B**) Gram staining result, and (**C**) 16S rDNA agarose gel electrophoresis of PCR amplified product of *Lactobacillus plantarum* CQPC11. M: 2000bp DNA Ladder; 0: negative control group; 11: *Lactobacillus plantarum* CQPC11.

The colony morphology of the strain is shown in Figure 1A. These colonies were mainly white or milky and had circular shapes with smooth edges and moist, smooth surfaces. Gram staining showed that the strain was stained positive and could be a lactic acid bacterium. Microscopic observation under 100× magnification indicated that this strain contained both long and short rod-shaped cells and that these did not undergo budding reproduction (Figure 1B). The results of agarose gel electrophoretic analysis of the 16S rDNA PCR products of the strain are shown in Figure 1C. No amplification was observed in the negative control, indicating that the PCRs were clear of contamination. A DNA fragment with the expected length of ~1,500 bp was amplified from this strain. BLAST analysis of the DNA sequencing results showed that this bacterium was *L. plantarum*. Its 16S rDNA shares 99% homology with that of known lactic acid bacteria listed in the GenBank database (GenBank Accession Number NC_010610.1). The strain was named *L. plantarum* CQPC11 (LP-CQPC11), patented (Patent No. is CGMCC 14960), and deposited in the CGMCC (China General Microbiological Culture Collection Center, Beijing, China).

### 2.2. Mouse Organ Indices

Table 1 shows that the thymus, brain, heart, liver, spleen, and kidney indices of the DGT (d-galactose-treated) group (the oxidation-induced aging group) significantly decreased compared to the normal group (*p* < 0.05), indicating that intraperitoneal injection of d-galactose caused tissue atrophy in these organs. However, the thymus, brain, heart, liver, spleen, and kidney indices of the two groups, namely, the group treated with lactic acid bacteria and that treated with vitamin C (DGT + Vc group), were significantly higher than those of the DGT group, indicating that tissue atrophy was mitigated in the three groups compared to the DGT group. LP-CQPC11 (DGT + LP-CQPC11 group) showed the best effects on suppressing tissue atrophy. Thus, LP-CQPC11 can effectively inhibit the reduction in organ indices and alleviate tissue atrophy due to d-galactose-induced oxidation and aging.

### 2.3. Levels of NO, SOD, GSH-Px, GSH, and MDA

Table 2, Table 3 and Table 4 show that the DGT mice had the highest NO and MDA levels, whereas the lowest SOD, GSH-Px, and GSH levels were observed in the serum, liver, and spleen. After treatment with *Lactobacillus delbruechii* subsp. *bulgaricus* (LDSB) (DGT + LDSB group), LP-CQPC11 (DGT + LP-CQPC11 group), or Vc (DGT + Vc group), the NO and MDA levels decreased, whereas those of SOD, GSH-Px, and GSH increased in the oxidation-induced aging mice. The NO, SOD, GSH-Px, GSH, and MDA levels of the group treated with LP-CQPC11 closely resembled those of the normal group. Thus, LP-CQPC11 was better than LDSB and Vc in terms of suppressing the oxidation- and aging-induced changes in NO, SOD, GSH-Px, GSH, and MDA levels in mice.

### 2.4. Pathological Observation of Mouse Liver and Spleen

**Figure 2:**
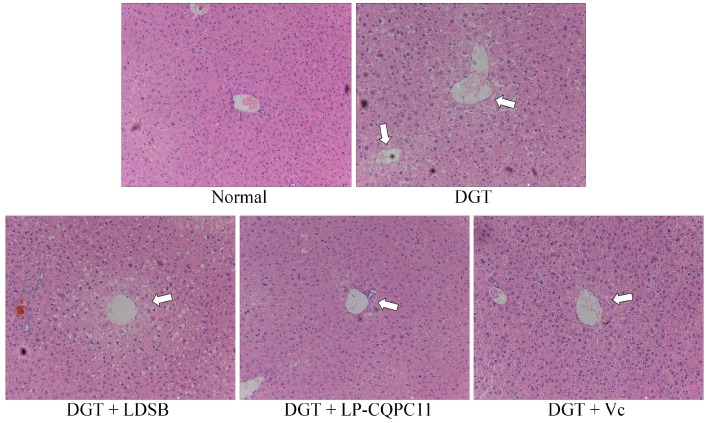
H&E pathological observation of liver in mice. Magnification 100× DGT: mice treated with d-galactose; DGT + LDSB: mice treated with d-galactose and *Lactobacillus delbruechii* subsp. *bulgaricus* (1.0 × 10^9^ CFU/kg); DGT + LP-CQPC11: mice treated with d-galactose and *Lactobacillus plantarum* CQPC11 (1.0 × 10^9^ CFU/kg); DGT + Vc: mice treated with d-galactose and vitamin C (200 mg/kg).

Histological micrographs of the mouse livers are shown in Figure 2. In the normal group, the mouse hepatocytes were well organized and showed normal morphology, uniform size, no signs of inflammation, and no obvious lesions. In the DGT group, the hepatocytes were disorganized and showed irregular morphology, loss of cell boundary, cell swelling, and widespread signs of inflammatory infiltration. Compared to the DGT group, the abnormalities of the hepatocytes in the DGT + LDSB, DGT + LP-CQPC11, and DGT + Vc groups were all reduced to certain levels. Most of the hepatocytes and the hepatic lobules of the DGT + LP-CQPC11 group were well organized. Hepatocytes in this group showed clear nuclear structure, nearly normal morphology, and only occasional cell infiltration. The results showed that LP-CQPC11 can alleviate the pathological changes in the liver tissues due to d-galactose-induced oxidation and aging in mice, and the effect of LP-CQPC11 is better than that of LDSB or Vc.

**Figure 3:**
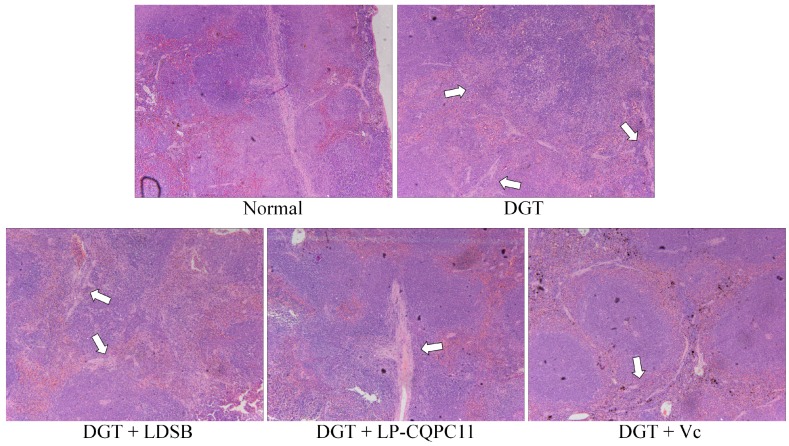
H&E pathological observation of spleen in mice. Magnification 100×. DGT: mice treated with d-galactose; DGT + LDSB: mice treated with d-galactose and *Lactobacillus delbruechii* subsp. *bulgaricus* (1.0 × 10^9^ CFU/kg); DGT + LP-CQPC11: mice treated with d-galactose and *Lactobacillus plantarum* CQPC11 (1.0 × 10^9^ CFU/kg); DGT + Vc: mice treated with d-galactose and vitamin C (200 mg/kg).

The histological micrographs of the spleen are shown in Figure 3. The normal group showed clear normal tissue morphology and cell organization. Compared to the normal group, the spleen lost its normal tissue morphology and cell organization, which was accompanied by dilated red pulp sinuses, a large number of red blood cells, reduced white pulp lymphocytes, narrowed red pulp cords, and reduced cell densities in the DGT group. Compared to the DGT group, spleen tissue morphology significantly improved with LDSB, LP-CQPC11, and Vc, which all showed clear boundaries between the red and white pulp, obvious germinal centers, and well organized cells. d-galactose-induced oxidation and aging showed the least detrimental effects on the spleen of the DGT + LP-CQPC11 group. Thus, LDSB, LP-CQPC11, and Vc can alleviate spleen damage caused by d-galactose-induced oxidation and aging in mouse models, with LP-CQPC11 showing the best effects.

### 2.5. Gene Expression Analysis of Mouse Liver

**Figure 4:**
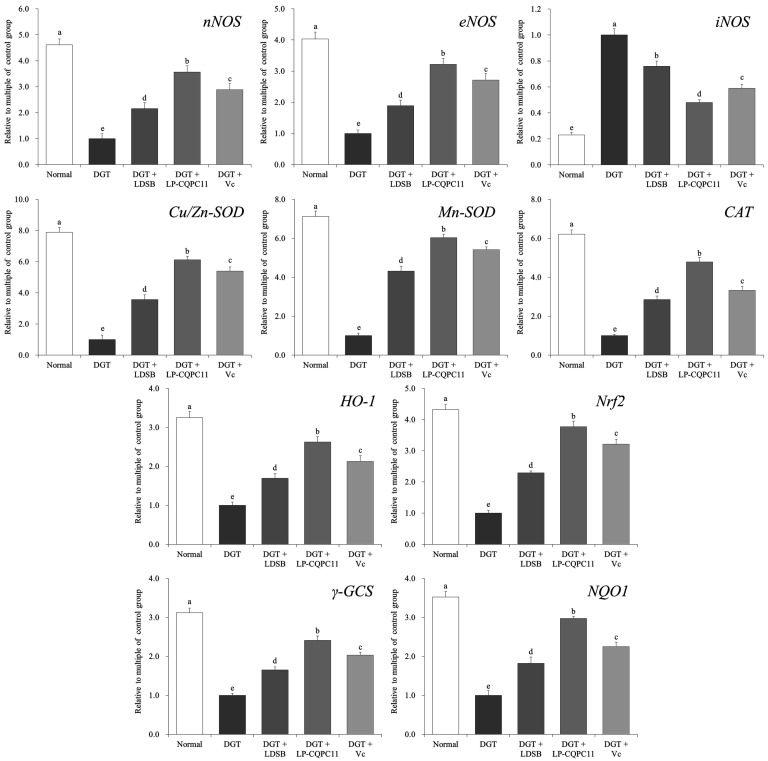
The mRNA expression in liver of mice. ^a–e^ The different letters mean that there are significant differences (*p* < 0.05) between every two groups according to Duncan’s multiple range test. DGT: mice treated with d-galactose; DGT + LDSB: mice treated with d-galactose and *Lactobacillus delbruechii* subsp. *bulgaricus* (1.0 × 10^9^ CFU/kg); DGT + LP-CQPC11: mice treated with d-galactose and *Lactobacillus plantarum* CQPC11 (1.0 × 10^9^ CFU/kg); DGT + Vc: mice treated with d-galactose and vitamin C (200 mg/kg).

Figure 4 shows that the liver of the normal group of mice exhibited the highest mRNA levels of *nNOS*, *eNOS*, *Cu/Zn-SOD*, *Mn-SOD*, *CAT*, *HO-1*, *Nrf2*, γ*-GCS*, and *NQO1*, and the lowest mRNA level of *iNOS*. In the liver of d-galactose-induced oxidation and aging mice, the expression levels of *nNOS*, *eNOS*, *Cu/Zn-SOD*, *Mn-SOD*, *CAT*, *HO-1*, *Nrf2*, γ*-GCS*, and *NQO1* significantly decreased (*p* < 0.05), whereas that of *iNOS* significantly increased (*p* < 0.05). The d-galactose-induced changes in the expression of these genes in the liver were significantly suppressed (*p* < 0.05) by LDSB, LP-CQPC11, and Vc, with LP-CQPC11 showing the best effects.

### 2.6. Gene Expression Analysis of Mouse Spleen

**Figure 5:**
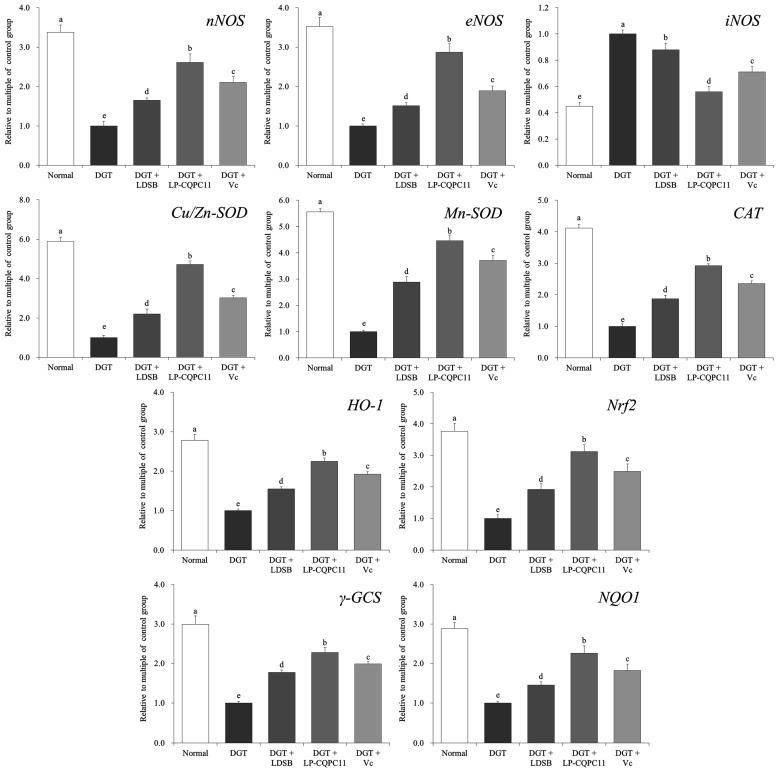
The mRNA expression in spleen of mice. ^a–e^ The different letters mean that there are significant differences (*p* < 0.05) between every two groups according to Duncan’s multiple range test. DGT: mice treated with d-galactose; DGT + LDSB: mice treated with d-galactose and *Lactobacillus delbruechii* subsp. *bulgaricus* (1.0 × 10^9^ CFU/kg); DGT + LP-CQPC11: mice treated with d-galactose and *Lactobacillus plantarum* CQPC11 (1.0 × 10^9^ CFU/kg); DGT + Vc: mice treated with d-galactose and vitamin C (200 mg/kg).

The expression patterns of the aforementioned genes in the spleen of each group were similar to those in the liver (Figure 5). The DGT mice showed the highest expression levels of *iNOS*, whereas the lowest expression levels of the other genes were observed in the spleen. After LP-CQPC11 was applied to the mice, the expression levels of these genes in the spleen were nearly restored to those of the normal mice. The spleen expression levels of *nNOS*, *eNOS*, *Cu/Zn-SOD*, *Mn-SOD*, *CAT*, *HO-1*, *Nrf2*, γ*-GCS*, and *NQO1* were higher in the DGT + LP-CQPC11 mice than in the DGT + LDSB and DGT + Vc mice, but the spleen *iNOS* expression level was lower in the DGT + LP-CQPC11 mice than in the DGT + LDSB and DGT + Vc mice. Thus, LP-CQPC11 could significantly suppress (*p* < 0.05) d-galactose-induced gene expression changes in mouse spleen.

### 2.7. Mouse Liver Protein Level Analysis

**Figure 6:**
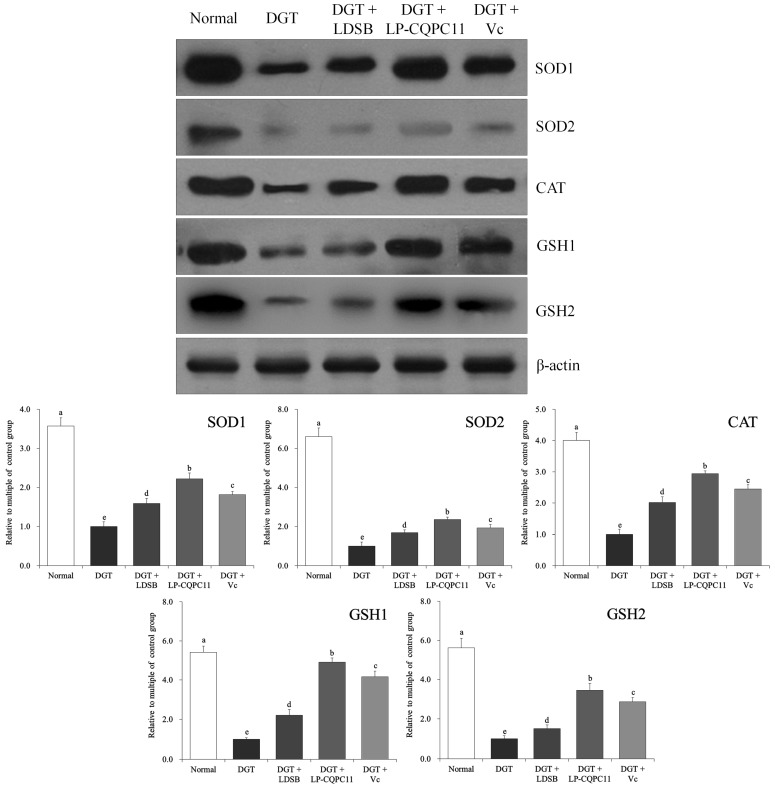
The protein expression in liver of mice. ^a–e^ The different letters mean that there are significant differences (*p* < 0.05) between every two groups according to Duncan’s multiple range test. DGT: mice treated with d-galactose; DGT + LDSB: mice treated with d-galactose and *Lactobacillus delbruechii* subsp. *bulgaricus* (1.0 × 10^9^ CFU/kg); DGT + LP-CQPC11: mice treated with d-galactose and *Lactobacillus plantarum* CQPC11 (1.0 × 10^9^ CFU/kg); DGT + Vc: mice treated with d-galactose and vitamin C (200 mg/kg).

Among all the groups of mice, the DGT group showed the highest—whereas the normal group showed the lowest—SOD1 (*Cu/Zn-SOD*), SOD2 (*Mn-SOD*), CAT, GSH1 (c-glutamylcysteine synthetase), and GSH2 (glutathione synthetase) protein levels in the liver (Figure 6). The liver expression levels of these proteins in the DGT + LP-CQPC11 group were lower than in the normal group, but significantly higher (*p* < 0.05) than in the DGT + LDSB and DGT + Vc groups.

### 2.8. Mouse Spleen Protein Level Analysis

**Figure 7:**
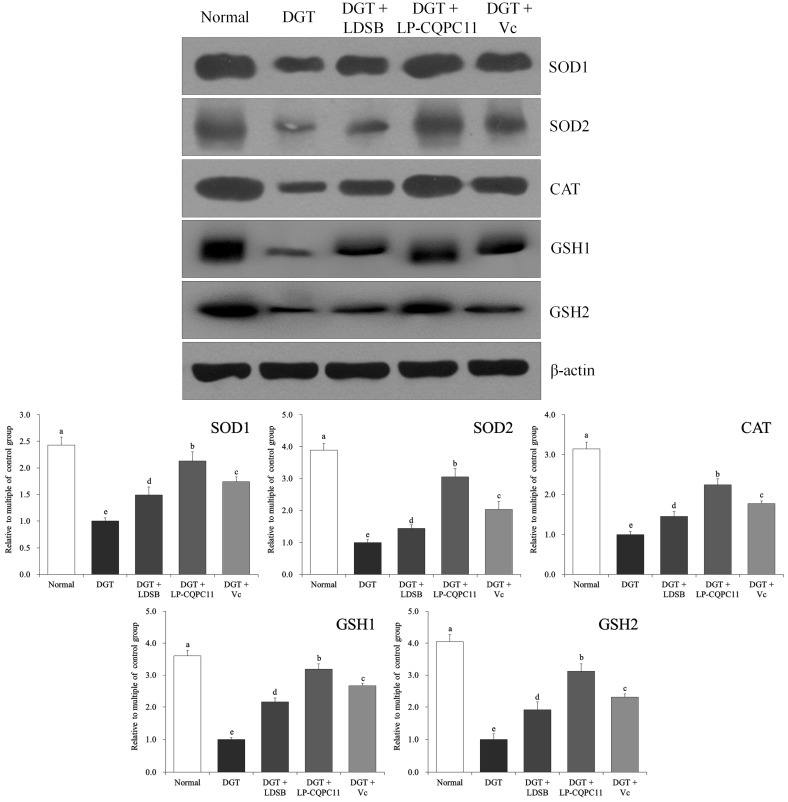
The protein expression in spleen of mice. ^a–e^ The different letters mean that there are significant differences (*p* < 0.05) between every two groups according to Duncan’s multiple range test. DGT: mice treated with d-galactose; DGT + LDSB: mice treated with d-galactose and *Lactobacillus delbruechii* subsp. *bulgaricus* (1.0 × 10^9^ CFU/kg); DGT + LP-CQPC11: mice treated with d-galactose and *Lactobacillus plantarum* CQPC11 (1.0 × 10^9^ CFU/kg); DGT + Vc: mice treated with d-galactose and vitamin C (200 mg/kg).

The protein expression levels of SOD1, SOD2, CAT, GSH1, and GSH2 in the spleen tissues were significantly higher (*p* < 0.05) in the normal group than in the other groups (Figure 7). LP-CQPC11 can significantly suppress the d-galactose-induced reduction in the SOD1, SOD2, CAT, GSH1, and GSH2 protein expression levels in the mouse spleen tissues. The experimental results showed that the protein expression levels of SOD1, SOD2, CAT, GSH1, and GSH2 in the spleen tissues were significantly higher (*p* < 0.05) in the DGT + LP-CQPC11 group than in the DGT + Vc and the DGT + LDSB groups.

## 3. Discussion

Changes in organ size can directly reflect aging of the body. When the body ages, the thymus and brain exhibit more profound atrophy than other organs. Thymus aging is the leading cause of immune system aging, and brain aging is one of the major characteristics of whole body aging [16]. The liver and kidney are two important metabolic organs and the decline in their size directly affects the body’s metabolism [17]. The spleen plays an important role in the immune system, and a reduction in its size is a sign of atrophy which can reduce its immune function [18]. The results of this study showed that oxidation-induced aging caused a decline in the organ indices in mice and that this decline can be effectively restored by LP-CQPC11 so that organ indices remain similar to those of normal mice.

NO is produced by NOS through the oxidization of l-arginine. Because the half-life of NO is very short, most of the studies of NO are based on the activity of NOS [19]. Three types of NOSs are involved in NO-mediated physiological or pathological processes, namely, nNOS, eNOS, and iNOS. NO is a typical free radical that can traverse the cell membrane freely and is highly oxidative. Under physiological conditions, NO generally conducts its biological functions by acting on soluble guanylate cyclase, but excessive amounts of NO can synergize with O^2−^ and cause cell damage [20]. NO and O^2−^ form ONOO^−^, which inactivates *Mn-SOD* in the mitochondria to promote the generation of free radicals, thus inducing oxidation and aging [21]. Under physiological conditions, nNOS and eNOS are expressed, but not iNOS, with nNOS being the most widely expressed one in tissues. The downregulation of the nNOS protein decreases the activity and the survival rate of the developing cerebellar granule cells cultured in vitro. nNOS can also promote the survival of mature cerebellar granule cells and may be related to aging [22]. eNOS can activate the soluble guanylate cyclase in vascular smooth muscle cells to increase intracellular cAMP concentration, thereby expanding blood vessels, inhibiting the adhesion and aggregation of platelet and leukocyte, protecting neurons, and antagonizing aging [23]. iNOS is mainly found in macrophages, inflammatory neutrophils, vascular smooth muscle cells, endothelial cells, microglia, and star cells. It continuously produces NO until the substrate is depleted. Excess amount of NO induces tissue damage through reactive nitrogen and the subsequent oxidative stress [24]. This study also confirmed that oxidation and aging induced by d-galactose can cause NOS dysregulation in the body. LP-CQPC11 can inhibit this dysregulation and restore the mRNA levels of nNOS, eNOS, and iNOS to normal, thus effectively preventing oxidation-induced aging.

GSH-Px is an important peroxidase widely present in the body. The main biological function of GSH-Px is to reduce lipid hydroperoxides, and high levels of GSH-Px in tissues can quickly remove H_2_O_2_. Under pathophysiological conditions, reactive oxygen species such as • OH induce lipid peroxidation, which directly damages biomembranes, and produce lipid hydroperoxide that reacts with proteins and nucleic acids, thus causing widespread tissue damage [25,26]. MDA is a lipid peroxide. Lipid peroxidation converts active oxygen into active chemical agents (i.e., non-radical lipid decomposition products), as well as amplifies the oxidative reaction of active oxygen by chain or branching chain reactions. Oxygen free radicals cause cell damage through both the peroxidation of polyunsaturated fatty acids in biomembranes and the decomposition products of lipid hydroperoxides. Therefore, the amount of MDA is often used to indicate lipid peroxidation in the body as well as the extent of cellular damage [27]. This study also confirmed that GSH-Px and MDA are directly related to oxidation-induced aging in mice, and LP-CQPC11 can antagonize oxidation-induced aging by increasing GSH-Px and lowering MDA levels.

As a highly efficient scavenger of oxygen and nitrogen free radicals, GSH plays an important role in maintaining cell redox status and tissue homeostasis. It is also involved in the regulation of immunity, extracellular matrix remodeling, apoptosis, and mitochondrial respiration. It can also be used as an intermediate for storing and transferring cysteine. As an antioxidant, it reacts with free radicals to produce an oxidized form, thereby protecting tissues and cells from attack by free radicals [28]. In the biosynthesis pathway of GSH, glutamate and cysteine are converted into γ-glutamylcysteine under the catalysis of GSH1, and then this intermediate product and glycine are used by GSH2 to synthesize GSH. GSH1 and GSH2 are both the key rate-limiting enzymes of GSH biosynthesis and their activities are controlled by feedback regulations [29]. γ-GCS is also a rate-limiting enzyme of GSH synthesis, which determines the level of GSH in vivo and is an important antioxidant factor. Meanwhile, studies have shown that the enhancer of γ-GCS contains Nrf2 binding sequence and is regulated by Nrf2. Nrf2 are positively correlated with γ-GCS mRNA and protein levels, and the knockout of Nrf2 resulted in the decreased expression of two subunits of γ-GCS in mouse fibroblasts and hepatocytes [30]. NQO1 is a protective reductase that protects cells from oxidation and aging induced by quinonoid compounds. As a target gene of Nrf2, NQO1 expression is enhanced to protect cells when Nrf2 is activated [31]. Therefore, LP-CQPC11 can act through GSH1, GSH2, γ-GCS, and Nrf2 to upregulate GSH levels and prevent oxidation-induced aging.

SOD is an important antioxidant enzyme in living organisms. It has special physiological activity and is the primary enzyme for scavenging free radicals. SOD can protect cells from the damage caused by oxygen free radicals and repair free radical-induced cell damage [32]. O_2_^−^ is mainly produced in the process of oxygen consumption and is chemically active. At high concentration, O_2_^−^ destroys macromolecules and causes cell metabolism disorder. SOD catalyzes the dismutation of O_2_^−^, thereby converting O_2_^−^ into hydrogen peroxide. Although hydrogen peroxide is a harmful active oxygen radical, it can be easily converted into H_2_O by CAT and SOD, thus avoiding oxidative chain reaction and cell damage [33]. There are mainly two types of SOD: *Mn-SOD* in mitochondrial matrix and CuZn-SOD in the cytosol blood cells. When the production of O_2_^−^ exceeds the scavenge capability of the body, the balance between oxidation and anti-oxidation will be lost, eventually leading to oxidation-induced aging and senescence. *Mn-SOD* is a key barrier against mitochondrial oxidation-induced aging and can effectively remove O_2_^−^ [34]. Studies have shown that the atrophy of some tissues can lead to a decrease in the expression of *Mn-SOD*. Meanwhile, oxidation-induced aging or chemically induced aging can also greatly reduce *Cu/Zn-SOD* [35]. CAT is an important antioxidant enzyme in the body, which maintains a dynamic balance with SOD in the body. CAT can decompose hydrogen peroxide into water and oxygen, thus avoiding oxidative tissue damage [36]. Hydrogen free radicals (OH^−^) are the most active reactive oxygen species in the body, which react with every type of organic molecules in the cell and lead to body ageing. OH^−^ is derived from the precursor hydrogen peroxide, whereas CAT can reduce the concentration of hydrogen peroxide to delay aging [37]. In this study, the authors found that LP-CQPC11 can antagonize oxidation-induced aging by regulating the levels of antioxidants such as SOD and CAT in mice.

HO-1 is an important antioxidant enzyme that plays a key role in protecting cells against endogenously and exogenously derived noxious stimuli. The antioxidant function of HO-1 is related to its capability of catalyzing the degradation of heme to prevent a free heme-mediated oxidation reaction. In addition to HO-1, its enzymatic product bilirubin and CO are also antioxidants and can improve tissue microcirculation [38]. Oxidative reagents including H_2_O_2_ activate HO-1 through Nrf2. The Nrf2/HO-1 pathway is widely involved in the anti-oxidative response of brain, liver, kidney, and nervous system, and is one of the most important endogenous protection mechanisms of the body [39]. Studies have found that leucosides upregulate HO-1 through serine/threonine protein kinase (Akt) and extracellular regulatory protein kinase (ERK1/2)/Nrf2 signaling pathways, thereby clearing ROS and suppressing hepatotoxicity. In addition, studies have shown that active ingredients in some plants also prevent oxidative stress and reduce cytotoxicity through the Nrf2/HO-1 pathway [40]. The authors’ results also suggest that LP-CQPC11 upregulates Nrf2/HO-1 pathway to prevent d-galactose-induced oxidative stress and aging in mice.

## 4. Materials and Methods

### 4.1. Isolation and Identification of Lactic Acid Bacteria

Approximately 1 mL of brine collected from commercially available pickled cabbages in Chongqing, China was used to prepare serial dilutions of 10^−4^, 10^−5^, and 10^−6^-fold of the original solution using sterile saline. Approximately 100 µL of each dilution were respectively spread on agar plates and then cultured at 37 °C for 48 h, and then the morphology of the emerging colonies was examined. The colonies with different morphologies were picked and streaked onto new plates. After culturing for 48 h at 37 °C, single colonies of different morphologies were picked and streaked onto new plates. This culture process was repeated two or three times until single colonies of similar morphology were obtained. These colonies were inoculated in 5 mL of MRS (DeMan-Rogosa-Sharpe) liquid medium and cultured at 37 °C for 24 h. Then, 1 mL of the culture solution was transferred into a sterile centrifuge tube and centrifuged at 4000 rpm for 10 min. The supernatant was discarded, and the cells were re-suspended in sterile physiological saline for gram staining. Then, the strains were again cultured in MRS broth at 37 °C for 24 h, and then their DNA was extracted using a genomic DNA extraction kit. The genomic DNA was used to amplify 16S rDNA by PCR, and the products were examined by agarose gel electrophoresis. Approximately 1 μL of the upstream primer (Thermo Fisher Scientific, Inc., Waltham, MA, USA) 7F (5′-AGA GTT TGA TCC TGGCTC AG-3′), 1 μL of the downstream primer 495R (5′-CTA CGG CTA CCTTGT TAC GA-3′), 2.5 μL of a 10×Taq plus buffer, and 1 μL of template DNA were mixed to prepare the PCR reaction mixture. Sterile ddH_2_O was then added to the mixture to a final volume of 25 μL. Sterile ultrapure water was used as negative control. The amplification parameters were as follows: 94 °C for 5 min; followed by 29 cycles of 94 °C for 30 s, 55 °C for 30 s, 72 °C for 1 min; and a final 72 °C for 5 min. Then, 5 μL of the amplification products were loaded onto a 1.5% agarose gel and separated by electrophoresis at 110 V for 45 min. Finally, the amplified 16S rDNA fragments were sequenced (SimpliAmp Thermal Cycler, Thermo Fisher Scientific, Inc., Waltham, MA, USA) [41]. The sequencing results were used to search the GenBank database using BLAST (Basic Local Alignment Search Tool).

### 4.2. Animal Models of Oxidation-Induced Aging

A total of 50 six-week-old SPF KM (Kunming) mice (25 males and 25 females) were fed for one week to adapt to the experimental environment and then divided into five groups, which were named as the normal group, the DGT group, the DGT + *L. delbruechii* subsp. *bulgaricus* group (DGT + LDSB group), the DGT + *L. plantarum* CQPC11 group (DGT + LP-CQPC11), and the DGT + vitamin C group (DGT + Vc). Each group consisted of five males and five females. The DGT + LDSB group and the DGT + LP-CQPC11 group were intragastrically administered with LDSB and LP-CQPC11 daily at a concentration of 1.0 × 10^9^ CFU/kg for four weeks (3rd week to 6th week). The DGT + Vc group was intragastrically administered with Vc at a concentration of 200 mg/kg daily for four weeks (3rd week to 6th week). Then, except for the normal group, the other four groups of mice were intraperitoneally injected with d-galactose at a concentration of 120 mg/kg for six weeks (1st week to 6th week). At the same time as the d-galactose injections, the LB, LP-CQPC11, and Vc groups continued to receive LB, LP-CQPC11, and Vc daily for six weeks. Then, all mice were fasted for 24 h and sacrificed by cervical dislocation [42]. The heart, blood, and liver were obtained for subsequent experiments. Meanwhile, the organ indices of thymus, brain, heart, liver, spleen, and kidney were determined using the following equation: Organ index = Organ mass (g)/Mouse body mass (kg) × 100. The study was conducted in accordance with the Declaration of Helsinki, and the protocol was approved by the Ethics Committee of Chongqing Medical University (No. SYXK 2018-0003).

### 4.3. Measurement of NO, SOD, GSH-Px, and MDA Levels in the Serum and Liver

The obtained plasma was placed for 30 min, then the plasma centrifuged at 4000 rpm for 10 min, and the supernatant was used to measure the serum levels of NO, SOD, GSH-Px, and MDA of the mice according to the kit instructions. The 10% mouse liver homogenates were centrifuged at 4000 rpm for 10 min, and the supernatant was taken to determine NO, SOD, GSH-Px and MDA levels according to the kit instructions (Nanjing Jiancheng Bioengineering Institute, Nanjing, China).

### 4.4. Pathological Observation of Liver and Spleen Tissues

About 0.5 cm^2^ of the mouse liver and spleen tissues were removed and soaked in 10% formalin solution for 48 h. The tissues were dehydrated, cleared, soaked in wax, embedded, sliced, stained with HE, and then examined under a light microscope (BX43, Olympus, Tokyo, Japan).

### 4.5. Quantitative PCR (qPCR) Assay

Total RNA was extracted from the liver and spleen tissues of the mice using RNAzol and diluted to 1 μg/μL. Next, 5 μL of the diluted RNA samples were used as template to generate cDNAs by reverse transcription according to the instructions of the transcription kit. PCR reactions were performed in a total volume of 14 μL, which consisted of 2 μL of cDNAs, 10 μL of SYBR Green PCR Master Mix (Thermo Fisher Scientific, Inc., Waltham, MA, USA), and 1 μL of each primer (Table 5). The PCR parameters were as follows: 95 °C for 60 s; followed by 40 cycles of 95 °C, for 15 s, 55 °C for 30 s, and 72 °C for 35 s; and a final 95 °C for 30 s and 55 °C for 35 s. GAPDH was used as internal reference, and the 2−ΔΔCt method was used to calculate relative gene expression levels [43].

### 4.6. Western Blot Analysis

Approximately 100 mg of liver and spleen tissue samples were homogenized in 1 mL of RIPA buffer supplemented with 10 μL of PMSF, and then cleared by centrifugation at 12,000 rpm at 4 °C for 4 min. The supernatant was collected and its protein concentration was determined using a BCA (bicinchoninic acid) protein quantification kit. The proteins were diluted to a concentration of 50 μg/mL, mixed with a sample buffer at a ratio of 4:1, heated at 100 °C for 5 min, separated by SDS-PAGE (Thermo Fisher Scientific, Inc., Waltham, MA, USA), and transferred onto a PVDF membrane. The PVDF membrane was blocked with 5% skim milk in TBST buffer for 1 h. After blocking, the PVDF membrane was washed with TBST and incubated with primary antibodies (Thermo Fisher Scientific, Inc., Waltham, MA, USA) at 25 °C for 2 h. The PVDF membrane was then washed with TBST, incubated with secondary antibodies at 25 °C for 1 h, and finally incubated with Supersignal West Pico PLUS. The membrane was placed in the iBright FL1000 system (Thermo Fisher Scientific, Inc., Waltham, MA, USA) for visualization [44].

### 4.7. Statistical Analysis

The results of serum and tissue measurements were the averages of three independent experiments. Then, the SAS9.1 statistical software was used for data analysis, and one-way ANOVA according to Duncan’s multiple-range test was used to determine statistical significance [44].

## 5. Conclusions

Previous studies have shown that lactic acid bacteria can decompose and neutralize harmful chemicals in the intestines, clean the intestines, prevent constipation, and suppress cell senescence, thus slowing aging [45]. The novel LP-CQPC11 that was identified in this study also showed good anti-aging effects in the mouse model of oxidation-induced aging. LP-CQPC11 can regulate multiple pathways that are involved in oxidation-induced aging in mice to prevent d-galactose-induced oxidation and aging. LP-CQPC11 can restore several serum and tissue indices of oxidation-induced aging mice close to those of normal mice, with effects even better than the commercially used LDSB, demonstrating good probiotic potential. Meanwhile, the anti-oxidation-induced aging effect of LP-CQPC11 is also better than that of vitamin C. The authors’ results suggested that LP-CQPC11 was a high-quality lactic acid bacterium with anti-oxidation-induced aging effects and that it could be used for further development of probiotic products. However, the development of LP-CQPC11 into probiotic products requires further human studies, which should be the focus of future research, as well as the elucidation of the underlying mechanisms by which LP-CQPC11 exerts its function in humans.

## Figures and Tables

**Table 1:** Organ index of mice in each group (*n* = 10).

Group	Thymus Index	Brain Index	Cardiac Index	Liver Index	Spleen Index	Kidney Index
Normal	0.25 ± 0.02 ^a^	1.91 ± 0.04 ^a^	5.02 ± 0.06 ^a^	38.21 ± 0.52 ^a^	4.12 ± 0.13 ^a^	12.89 ± 0.39 ^a^
DGT	0.14 ± 0.02 ^d^	1.17 ± 0.07 ^d^	4.12 ± 0.05 ^e^	30.32 ± 1.32 ^e^	2.67 ± 0.28 ^e^	9.22 ± 0.27 ^e^
DGT + LDSB	0.18 ± 0.02 ^c^	1.48 ± 0.06 ^c^	4.35 ± 0.08 ^d^	33.15 ± 0.46 ^d^	3.28 ± 0.12 ^d^	10.03 ± 0.26 ^d^
DGT + LP-CQPC11	0.22 ± 0.01 ^b^	1.79 ± 0.06 ^b^	4.81 ± 0.07 ^b^	35.73 ± 0.67 ^b^	3.77 ± 0.15 ^b^	12.11 ± 0.20 ^b^
DGT + Vc	0.19 ± 0.02 ^c^	1.54 ± 0.07 ^c^	4.62 ± 0.07 ^c^	34.39 ± 0.47 ^c^	3.49 ± 0.11 ^c^	11.46 ± 0.25 ^c^

Values presented are the mean ± standard deviation (*n* = 10/group). ^a–^^d^ In the same column, the different letters mean that there are significant differences (*p* < 0.05) between every two groups, and the same letters mean that there is no significant difference (*p* > 0.05) between every two groups according to Duncan’s multiple range test. DGT: mice treated with d-galactose; DGT + LDSB: mice treated with d-galactose and *Lactobacillus delbruechii* subsp. *bulgaricus* (1.0 × 10^9^ CFU/kg); DGT + LP-CQPC11: mice treated with d-galactose and *Lactobacillus plantarum* CQPC11 (1.0 × 10^9^ CFU/kg); DGT + Vc: mice treated with d-galactose and vitamin C (200 mg/kg).

**Table 2:** The levels of NO, SOD, GSH-Px, GSH, and MDA in serum of mice (*n* = 10).

Group	NO (μmol/L)	SOD (U/mL)	GSH-Px (U/mL)	GSH (mg/L)	MDA (nmol/mL)
Normal	18.79 ± 0.45 ^e^	275.36 ± 8.71 ^a^	182.36 ± 7.87 ^a^	37.33 ± 0.52 ^a^	5.57 ± 0.35 ^e^
DGT	63.69 ± 1.12 ^a^	97.63 ± 8.16 ^e^	103.55 ± 6.89 ^e^	14.02 ± 1.13 ^e^	29.35 ± 1.03 ^a^
DGT + LDSB	42.72 ± 1.02 ^b^	203.56 ± 9.71 ^d^	119.71 ± 4.38 ^d^	18.33 ± 0.55 ^d^	20.45 ± 0.59 ^b^
DGT + LP-CQPC11	30.55 ± 0.67 ^d^	147.83 ± 7.89 ^b^	152.03 ± 5.88 ^b^	29.42 ± 0.63 ^b^	10.32 ± 0.38 ^d^
DGT + Vc	38.32 ± 0.66 ^c^	178.36 ± 6.25 ^c^	133.20 ± 6.34 ^c^	23.57 ± 0.61 ^c^	16.74 ± 0.68 ^c^

Values presented are the mean ± standard deviation (*n* = 10/group). ^a–^^e^ In the same column, the different letters mean that there are significant differences (*p* < 0.05) between every two groups according to Duncan’s multiple range test. DGT: mice treated with d-galactose; DGT + LDSB: mice treated with d-galactose and *Lactobacillus delbruechii* subsp. *bulgaricus* (1.0 × 10^9^ CFU/kg); DGT + LP-CQPC11: mice treated with d-galactose and *Lactobacillus plantarum* CQPC11 (1.0 × 10^9^ CFU/kg); DGT + Vc: mice treated with d-galactose and vitamin C (200 mg/kg).

**Table 3:** The levels of NO, SOD, GSH-Px, GSH, and MDA in liver of mice (*n* = 10).

Group	NO (μmol/gprot)	SOD (U/mgprot)	GSH-Px (U/mgprot)	GSH (mg/gprot)	MDA (nmol/mgprot)
Normal	3.21 ± 0.15 ^e^	95.18 ± 5.32 ^a^	163.21 ± 7.91 ^a^	8.32 ± 0.28 ^a^	2.11 ± 0.19 ^e^
DGT	9.45 ± 0.41 ^a^	31.02 ± 2.65 ^e^	91.03 ± 4.53 ^e^	2.65 ± 0.29 ^e^	9.39 ± 0.42 ^a^
DGT + LDSB	7.16 ± 0.33 ^b^	48.63 ± 4.61 ^d^	113.25 ± 5.33 ^d^	4.32 ± 0.25 ^d^	6.41 ± 0.33 ^b^
DGT + LP-CQPC11	4.85 ± 0.12 ^d^	74.36 ± 5.01 ^b^	140.73 ± 3.25 ^b^	6.12 ± 0.22 ^b^	3.89 ± 0.21 ^d^
DGT + Vc	5.74 ± 0.20 ^c^	60.87 ± 4.33 ^c^	127.18 ± 4.25 ^c^	5.39 ± 0.28 ^c^	4.77 ± 0.18 ^c^

Values presented are the mean ± standard deviation (*n* = 10/group). ^a–^^e^ In the same column, the different letters mean that there are significant differences (*p* < 0.05) between every two groups according to Duncan’s multiple range test. DGT: mice treated with d-galactose; DGT + LDSB: mice treated with d-galactose and *Lactobacillus delbruechii* subsp. *bulgaricus* (1.0 × 10^9^ CFU/kg); DGT + LP-CQPC11: mice treated with d-galactose and *Lactobacillus plantarum* CQPC11 (1.0 × 10^9^ CFU/kg); DGT + Vc: mice treated with d-galactose and vitamin C (200 mg/kg).

**Table 4:** The levels of NO, SOD, GSH-Px, GSH, and MDA in spleen of mice (*n* = 10).

Group	NO (μmol/gprot)	SOD (U/mgprot)	GSH-Px (U/mgprot)	GSH (mg/gprot)	MDA (nmol/mgprot)
Normal	1.49 ± 0.15 ^e^	70.87 ± 3.26 ^a^	115.36 ± 4.22 ^a^	5.89 ± 0.18 ^a^	1.03 ± 0.06 ^e^
DGT	7.41 ± 0.28 ^a^	22.36 ± 4.12 ^e^	44.31 ± 3.55 ^e^	1.52 ± 0.23 ^e^	4.24 ± 0.16 ^a^
DGT + LDSB	5.88 ± 0.24 ^c^	45.97 ± 3.87 ^d^	60.12 ± 2.88 ^d^	2.74 ± 0.21 ^d^	3.11 ± 0.15 ^b^
DGT + LP-CQPC11	2.28 ± 0.14 ^d^	61.25 ± 2.02 ^b^	87.71 ± 3.91 ^b^	4.12 ± 0.24 ^b^	1.62 ± 0.10 ^d^
DGT + Vc	3.31 ± 0.20 ^c^	51.88 ± 2.31 ^c^	69.78 ± 3.02 ^c^	3.65 ± 0.22 ^c^	2.41 ± 0.11 ^c^

Values presented are the mean ± standard deviation (*n* = 10/group). ^a–^^e^ In the same column, the different letters mean that there are significant differences (*p* < 0.05) between every two groups according to Duncan’s multiple range test. DGT: mice treated with d-galactose; DGT + LDSB: mice treated with d-galactose and *Lactobacillus delbruechii* subsp. *bulgaricus* (1.0 × 10^9^ CFU/kg); DGT + LP-CQPC11: mice treated with d-galactose and *Lactobacillus plantarum* CQPC11 (1.0 × 10^9^ CFU/kg); DGT + Vc: mice treated with d-galactose and vitamin C (200 mg/kg).

**Table 5:** Sequences of primers used in this study.

Gene Name	Sequence
*nNOS*	Forward: 5′-ACGGCAAACTGCACAAAGC-3′
	Reverse: 5′-CGTTCTCTGAATACGGGTTGTTG-3′
*eNOS*	Forward: 5′-TCAGCCATCACAGTGTTCCC-3′
	Reverse: 5′-ATAGCCCGCATAGCGTATCAG-3′
*iNOS*	Forward: 5′-GTTCTCAGCCCAACAATACAAGA-3′
	Reverse: 5′-GTGGACGGGTCGATGTCAC-3′
*Cu/Zn-SOD*	Forward: 5′-AACCAGTTGTGTTGTCAGGAC-3′
	Reverse: 5′-CCACCATGTTTCTTAGAGTGAGG-3′
*Mn-SOD*	Forward: 5′-CAGACCTGCCTTACGACTATGG-3′
	Reverse: 5′-CTCGGTGGCGTTGAGATTGTT-3′
*CAT*	Forward: 5′- GGAGGCGGGAACCCAATAG -3′
	Reverse: 5′-GTGTGCCATCTCGTCAGTGAA-3′
*HO-1*	Forward: 5′-ACAGATGGCGTCACTTCG-3′
	Reverse: 5′-TGAGGACCCACTGGAGGA-3′
*Nrf2*	Forward: 5′-CAGTGCTCCTATGCGTGAA-3′
	Reverse: 5′-GCGGCTTGAATGTTTGTC-3′
*γ-GCS*	Forward: 5′-GCACATCTACCACGCAGTCA-3′
	Reverse: 5′-CAGAGTCTCAAGAACATCGCC-3′
*NQO1*	Forward: 5′-CTTTAGGGTCGTCTTGGC-3′
	Reverse: 5′-CAATCAGGGCTCTTCTCG-3′
*GAPDH*	Forward: 5′-AGGTCGGTGTGAACGGATTTG-3′
	Reverse: 5′-GGGGTCGTTGATGGCAACA-3′

*nNOS*: neuronal nitric oxide synthase; *eNOS*: endothelial nitric oxide synthase; *iNOS*: inducible nitric oxide synthase; *Cu/Zn-SOD*: cuprozinc-superoxide dismutase; *Mn-SOD*: manganese superoxide dismutase; *CAT*: catalase; *HO-1*: heme oxygenase-1; *Nrf2*: nuclear factor-erythroid 2 related factor 2; *γ-GCS*: γ-glutamylcysteine synthetase; *NQO1*: NAD(P)H dehydrogenase [quinone] 1; *GAPDH*: glyceraldehyde-3-phosphate dehydrogenase.
